# Comparison of the eSwab collection and transportation system to an amies gel transystem for Gram stain of clinical specimens

**DOI:** 10.1186/1756-0500-2-244

**Published:** 2009-12-10

**Authors:** Carla Fontana, Marco Favaro, Dolores Limongi, Jana Pivonkova, Cartesio Favalli

**Affiliations:** 1 Department of Experimental Medicine and Biochemical Sciences, "Tor Vergata" University of Rome, Via Montpellier 1, 00133 Rome, Italy; 2 Clinical Microbiology Laboratories, Polyclinic of Tor Vergata, Viale Oxford 81, 00133 Rome, Italy

## Abstract

**Background:**

The first step in routine microbiology laboratory procedures is the collection and safe transportation of swab samples. This can be accomplished using ESwab Collection and Transport System (Copan Italia, Brescia -Italy). The aim of the present study was to compare the results of microscopic examination of gram stain smears prepared directly from clinical specimens, collected and transported in the ESwab, with those obtained using Amies Agar gel Transystem without charcoal (Copan).

**Findings:**

Specimens were collected from 80 patients (32 vaginal swabs, 27 cervical swabs, 11 urethral swabs and 10 wound swabs). Two swabs were in random order collected from each patient, one using the conventional Amies gel Transystem, the other using ESwab. One slide was prepared for each specimen using the conventional swab and two sets of slides were prepared from the specimens collected with the ESwab: one using 100 μl and one using 50 μl of the Amies medium. All slides were gram stained using an automated Gram stainer. Microscopic examination of 240 slides (80 with conventional and 160 with ESwab) showed that the quality of smear preparation from the ESwab system, allowed for easier identification of human cells and identification of greater number of microorganisms. Microscopic examination of additional slides prepared from ESwab at 24 or 72 hours after initial collection were equivalent to those prepared when received in the laboratory within 2 hours of collection.

**Conclusion:**

Microscopic examination performed using ESwab, especially when preparing the slides with 100 μl, shows superior results to those obtained using the Amies gel Transystem.

## Background

Microscopic examination is an important initial diagnostic test in the processing of specimens in the clinical microbiology laboratory [1-3]. The Gram stain is used to classify bacteria on the basis of form, size, cellular morphology, and Gram reactions. The Gram stain can be a critical test for the rapid presumptive diagnosis of infectious agents and serve to assessment of the quality of clinical specimens [1-6]. The timely report of a Gram stain result gives the physician useful information and allows the laboratory several options to triage specimens. The preparation and analysis of a Gram stain is a procedure which requires experience in order for a correct result to be reported. A good specimen collection system is necessary especially if the Gram slide is prepared directly at the time of collection [4,7,8]. Appropriate specimen collection and transportation are essential for accurate laboratory diagnosis. Because of their convenience, swab systems with transport media are often used to collect and transport specimens of various types. Amies gel Transystem together with a sterile rayon swab are used extensively to collect and submit clinical specimens to the laboratory. The Copan ESwab collection and transport system is a more recent collection and transportation system which incorporates a modified Liquid Amies transport medium and a flocked swab. The liquid Amies sustains the viability of a variety of organisms including aerobes, anaerobes and fastidious bacteria [9].

Van Horn *et al *have recently evaluated the ESwab system, on the basis of the CLSI acceptance criteria, concluding that it's an acceptable swab transport system for maintaining viability of both aerobes and anaerobes [9]. To our knowledge no studies have been performed in order to evaluate the ESwab system with regards to the Gram-stain. The objective was to compare smears of clinical specimens collected and transported in the ESwab system to clinical specimens collected and transported in Amies gel Transystem for Gram stain.

## Methods

**Figure 1:**
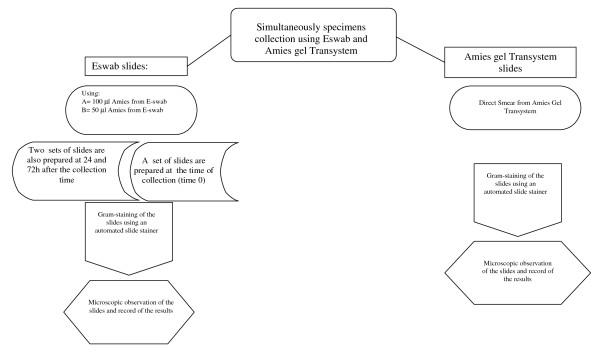
**Flow chart of slides preparation**.

Specimens A total of 80 patients (32 vaginal swabs, 27 cervical swabs, 11 urethral swabs and 10 wound swabs) were sampled. Two swabs were simultaneously collected from each patient, the first one using the conventional rayon swab of the Amies gel Transystem, the other using the nylon flocked swab of the ESwab system (see figure 1). The specimens were collected by standard practice.

### Preparation of slides using ESwab

Once a swab sample was collected, it was placed immediately into the ESwab transport tube containing transport medium. Specimens were transported directly to the laboratory (within 2 h). The ESwab specimen tube was briefly vortexed then the swab was removed according to the manufacturer's instructions. The Eswab liquid Amies was used immediately to prepare two slides for microscopic examination. The first slide was prepared using 100 μl of liquid Amies medium from the ESwab tube, the second using 50 μl. The aliquot of liquid Amies medium was spread onto the surface of the slide with the help of a second slide (using a technique similar to that employed for making blood smears). The slides were air dried and fixed with the use of 70-95% methanol for 1 minute (avoiding heat which alters cell morphology and makes organisms more susceptible to over-decolourization). The methanol was drained off, and the slides were air-dried.

### Preparation of slides using Amies gel Transystem

The slides were prepared directly by rolling and smearing the swab on the slide. The slides were air dried and fixed with 70-95% methanol for 1 minute. The methanol was then drained off, and the slides were air-dried.

### Gram-stain technique and Microscopic observation

All the slides were Gram-stained using the AEROSPRAY^® ^MICROBIOLOGY SLIDE STAINER (Delcon Italia, Arcore-Milan Italy) following the instructions of the manufacturer. Quality control procedures were performed to help ensure that the information reported was accurate, reliable and reproducible and for evaluating Gram stain reagents and staining techniques. Each stain run included a commercially available BD BBL™ Gram Slide Control (Becton Dickinson, Becton Drive Franklin Lakes, NJ, USA). A few drops of immersion oil were spread over the smear, which was then examined at × 1000 magnification. The entire smear was observed for all slides. To ensure accuracy of interpretation, Gram stains were viewed by two different competent persons and reviewed by a supervisor. Gram stain from genital specimens was scored on the grading system of Ison et al, while Gram stain from wound swabs was evaluated following Q score method of Matkosky et al [10,11].

### Maintenance of morphology of human cells and microorganisms in ESwab medium

In order to evaluate the impact of a delayed microscopic examination of samples collected using the ESwab system, two sets of slides were prepared at 24 and 72 h after the collection time (during this time the ESwab were stored at 5°C). The slides were prepared, stained and observed as reported above.

### Statistical analysis

Comparison of proportion was performed with a two-sided Fisher's exact test. Differences were considered significant at a *p *value of ≤ 0.05 [12].

### Document Ethical Aprroval

17/03/2008 by Ethical Committee (Prof P. Fucci)

## Results and Discussion

**Figure 2:**
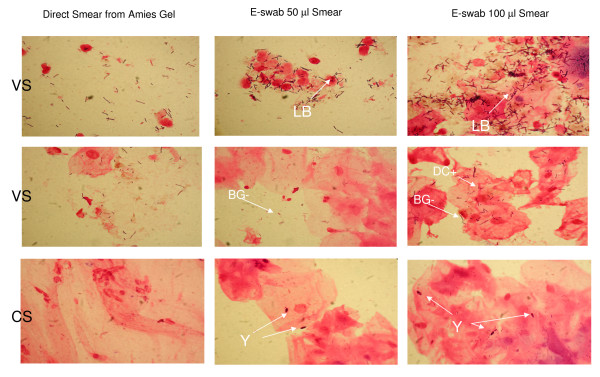
**Comparison of some microscopic observations**. LB = Lactobacilli; BG-: Gram-negative bacilli; DC+: Gram-positive diplococci; Y: yeast cell; VS: Vaginal Swab; CS: Cervical swab.

The microscopic examination of 240 slides from 80 specimens collected from 80 patients showed that the quality of smear preparation from the ESwab system, particularly those prepared using 100 μl of Amies medium, was superior to those obtained using the Amies gel Transystem. The overall results were reported in table 1. The ESwab slides prepared using 100 μl of Amies medium (22/80) demonstrated better details of epithelial cells, leukocytes, and red blood cells (see table 2). They also contained bacteria (particularly Gram-negative bacilli and Gram-positive diplococci) or fungi not present in the Amies slides (29/80) (table 2 and figure 2). On the basis of these findings the score of 27 genital specimens changed from I to II or III. While Q score, applied for wounds, moved towards a positive value due to the observation of polymorphonuclear cells (lacking in Amies gel slides). Culture performed on the specimens (data not shown) confirmed the microscopic evidence observed in Eswab slides (e.g. yeast: corresponded to the growth of *Candida *spp.; Diplococci: corresponded to the culture of *Streptococcus agalactiae*; Gram-negative bacilli: corresponded to the growth of some enterobacteria, and so on) and no false negative results were recorded using Eswab slides. Differences among the ESwab slides and Amies gel slides were statistically significant, *p *value ≤ 0.04 [12]. While no significant differences were observed comparing the microscopic exams of ESwab slides, those prepared using 100 μl of liquid Amies medium were better than those prepared with 50 μl. In addition, the slides prepared from the samples collected in the ESwab exhibit a very good preservation of cells. Comparing the results of fresh ESwab slides with those after 24 and 72 h storage, no significant differences were observed (table 1). An ideal swab system must have the ability to absorb organisms from the site of infection, to maintain the viability of organisms during transport and to allow the release of organisms from the swab onto a slide or appropriate culture media. Our findings showed that the slides prepared from ESwab system were observed to demonstrate more human cell and bacterial species than the traditional Amies gel system. These differences are mainly attributed to the flocked swab collection device. The flocked swab collected bacteria by capillary action whereas the traditional swab absorbed bacteria into the cotton, dacron or rayon fiber matrix [13]. The flocked swab demonstrates a superior absorption and release (onto the slide surface) as evidenced by the significantly higher counts of human cells as well as microbes [13,14]. Other studies have shown that the ESwab system also preserves excellent viability of several organisms [9,15]. With the ESwab system the 1 ml of eluted homogeneous liquid suspension can be used to prepare the Gram stain and inoculate agar plates manually or with an automated instrument, with a more standardized sample volume than plain swabs. The remaining portion of the specimen may also be frozen and used later if required. Sub-heading for this section

**Table 1:** Microscopic examination of ESwab gram-slides versus Amies gel slides.

Specimen types	Results are expressed as: no. of slides presenting differences in microscopic observation of human cells and/or of microbial elements/no. of samples tested		
	
(no.)	ESwab Volumes for slides preparation		
		
	100 μl^§^	50 μl^§^	Amies Gel slides
Vaginal Swab (32)	32/32	26/32	16/32
Cervical Swab (27)	27/27	25/27	15/27
Urethral Swab (11)	11/11	11/11	8/11
Wound Swab (10)	10/10	10/10	7/10

Total (80)	80/80 (100%)	72/80(90%)	46/80 (57.5%)

P value		P = 0.16	P = 0.04

§ = the results were the same even after 24 and 72 h storage.

**Table 2:** Microorganisms and human cells present in the microscopic examination of ESwab slides prepared from ESwab (using 100 μl of Amies medium) which were not in the Amies gel slides.

Specimens Type (no.)					Total
	**Vaginal Swab** **(32)**	**Cervical Swab (27)**	**Urethral Swab (11)**	**Wound Swab (10)**	**80**

***Microorganisms present in microscopic examination***					
Yeast Cells	5	3			
Gram-negative diplococci			1		
Gram-positive diplococci/streptococci	5	3			
*Trichomonas *spp.	1				
Clue cells due to Gram-negative coccobacilli		1	1		
*Mobilincus *spp	1				
Gram-negative bacilli	3	3	1	1	

**Subtotal**	**15**	**10**	**3**	**1**	**29/80 (36,25%)**

***Human cells present in the microscopic examination***					
> Epithelial cells per field of view	6				
5-6 leucocytes per field of view	1	3			
Numerous leucocytes (20-30 per field of view)	3	3	1	2	
Blood Cells		2		1	

**Subtotal**	**10**	**8**	**1**	**3**	**22/89 (27,5%)**

## List of Abbreviations

LB: Lactobacilli; BG-: Gram-negative bacilli; DC+: Gram-positive diplococci; Y: yeast cell; VS: Vaginal Swab; CS: Cervical swab.

## Competing interests

The authors declare that they have no competing interests.

## Authors' contributions

CF and MF contributed to the conception of the study, in data analysis and are also involved in drafting the manuscript. FC contributed to the review of the study and data analysis. DL and JP contributed in acquisition and interpretation of data.

All authors approved the final version of the manuscript.
